# Classification of endonasal HHT lesions using digital microscopy

**DOI:** 10.1186/s13023-021-01801-9

**Published:** 2021-04-17

**Authors:** F. Haubner, A. Schneider, H. Schinke, M. Bertlich, B. G. Weiss, M. Canis, F. Kashani

**Affiliations:** 1 Department of Otorhinolaryngology, Head and Neck Surgery, University Hospital, LMU Munich, Marchioninistrasse 15, 81377 Munich, Germany; 2 ARRI Medical GmbH, Munich, Germany

**Keywords:** Digital microscopy, Epistaxis, HHT, Morbus Osler, Laser

## Abstract

**Background:**

Recurrent spontaneous epistaxis is the most common clinical manifestation and the most debilitating symptom in hereditary haemorrhagic telangiectasia (HHT) patients. To this date, there exist only a classification of HHT patients by different genetic mutations. There is no standard classification for the mucocutaneous endonasal manifestations of HHT. The aim of the present study was to document the variety of endonasal HHT lesions using digital microscopy and to propose a clinical classification.

**Methods:**

We recorded the endonasal HHT lesions of 28 patients using a digital microscope. We reconstructed the 3D images und videos recorded by digital microscope afterwards and classified the endonasal lesions of HHT in two classes: Grade A, presence of only flat telangiectasias in the mucosa level and Grade B, (additional) presence of raised berry or wart-like telangiectasia spots. We investigated also Haemoglobin level by routine laboratory procedures, plasma VEGF level by ELISA, Severity of epistaxis by epistaxis severity score (ESS) and quality of life by a linear visual analogue scale (VAS).

**Results:**

We found a higher quality of life and a lower severity of epistaxis in Grade A patients in comparison to Grade B patients. No difference in plasma VEGF level and in Haemoglobin between Grad A patients and Grade B patients could be detected. Plasma VEGF levels showed no gender specific differences. It could also not be correlated to the extranasal manifestation.

**Conclusion:**

The classification for endonasal manifestation of HHT proposed in this study indicates severity of epistaxis und quality of life. Digital microscopy with the ability of 3D reconstruction of images presents a useful tool for such classifications. The classification of endonasal HHT lesions using digital microscopy allows to evaluate the dynamic of HHT lesions in the course of time independent of examiner. This allows also to evaluate the efficacy of the different treatment modalities by dynamic of HHT lesions. Moreover digital microscopy is very beneficial in academic teaching of rare diseases.

**Supplementary Information:**

The online version contains supplementary material available at 10.1186/s13023-021-01801-9.

## Introduction

Hereditary Haemorrhagic Telangiectasia (HHT) is an autosomal dominant vascular disorder with an estimated prevalence of 1 in 5000 to 10,000 individuals worldwide [1, 2]. Mutations in several genes of the transforming growth factor (TGF)-β superfamily pathway in patients with HHT results in unregulated vessel wall remodelling within the mucosa and viscera [3–7]. This leads to the prolongated activation phase of angiogenesis, where angiogenic factors, such as vascular endothelial growth factor (VEGF), are overexpressed and play a potential role in angiodysplasia [8, 9]. Angiodysplastic lesions show a loss of elastic elements, resulting in markedly dilated and convoluted postcapillary venules, which often have a direct connection to dilated arterioles [8, 9]. HHT is associated with the development of vascular malformations in various organs, such as brain, lung, liver, skin and the mucous membranes, especially within the upper aerodigestive tract. The vascular malformations may lead to recurrent and even life-threatening haemorrhage [10]. Recurrent spontaneous epistaxis is the most common clinical manifestation and the most debilitating symptom in up to 95 % of HHT patients [11]. It has been shown, that the majority of endonasal telangiectasias are found in the periphery of the supplying vessels of nasal mucosa and in the areas with a higher mechanical stress, especially where the laminar respiratory airflow changes into a turbulent flow [12]. While the most endonasal telangiectasias are located within the anterior nasal cavity, manifestations can also be found in the posterior parts of the nasal cavity and in the nasopharynx [12]. The morphology of nasal telangiectasia represents a strong variation from small punctate lesion to large exophytic spots. Lesions can be scattered and isolated or be confluent [12, 13]. Morphology and distribution of endonasal telangiectasias and their clinical correlations have rarely been the topic of systematic studies. To this date, there is no standard classification for the mucocutaneous endonasal manifestations of HHT.

A variety of treatment modalities is available for either acute or chronic management of epistaxis in HHT. Anti-VEGF (Bevacizumab) treatment is one of the treatment modalities that has recently been under intense investigation. It has been shown, that plasma VEGF levels are elevated significantly in HHT patients [14, 15]. It has also found to be a positive correlation of plasma levels with the degree of bleeding reductions after local submucosal bevacizumab treatment [16].

The aim of this study was evaluating 3D picture and video documentation of endonasal HHT lesions using a digital 3D microscope to propose a classification of endonasal HHT manifestation, which could represent a clinical correlation to the severity of nasal symptoms in HHT. In addition, we investigated the correlation between plasma VEGF level and the severity of nasal symptoms, as well as presence of extranasal manifestation in HHT-patients.

## Materials and methods

### Ethics

This study was approved by the local ethics committee under the ongoing file number 19-094. The ethics committee waived the need for informed consent since the study was limited to sole data collection during regular standard practice; there were no changes in treatment caused by this study.

### Patient selection, recruitment and study design

The study represents a retrospective chart review conducted in a tertiary referral center with a specialization for vascular malformations and HHT. All patients affected with HHT according to the Curaçao criteria [17] and presenting visible endonasal manifestations of HHT with recurrent epistaxis were enrolled into the study. All patients were treated with a blue light laser system (TruBlue™, A.R.C. Laser GmbH, Nuremberg, Germany) at 445 nm and 1.4 W using a 300 μm bare fibre under microscopic view. The analysis was performed for patients treated at our institution between November 2019 an May 2020.

### Imaging and classification of endonasal HHT-Manifestation

**Fig. 1:**
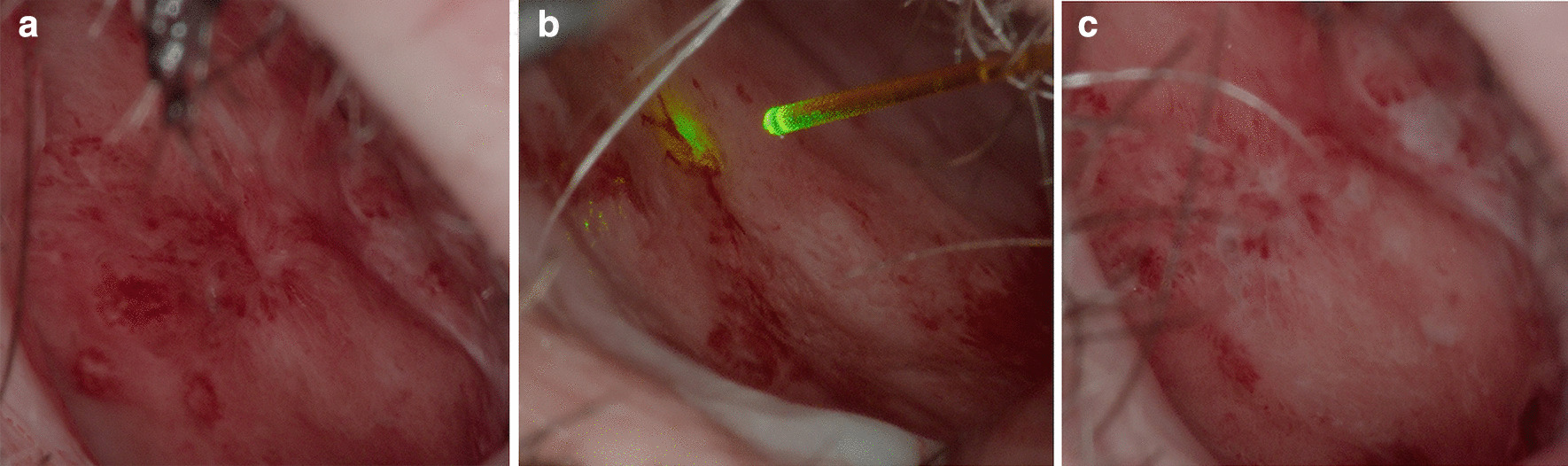
Treatment of endonasal telangiectasias in HHT using a 445 nm laser system (TruBlue™, ARC, Nuremberg, Germany); **a** before treatment, **b** during treatment, **c** after treatment

Imaging was done with a fully digital operating microscope delivering high-definition output streams in 3D (ARRISCOPE 1.1, ARRI Medical GmbH, Munich, Germany). Images were recorded before and after the treatment with a resolution of 1920 × 1080 px and 16-bit color depth in a Tagged Image File Format (TIF). At the same time, videos were recorded in the same resolution with a H.264 compression for later evaluation. To prevent saturation of the imaging sensor in the digital microscope during application of the laser pulse, two notch filters with a diameter of 22 mm and a blocking bandwidth of 445 nm (A.R.C. Laser GmbH, Nuremberg, Germany) were installed in the two imaging channels for the left and right image at the achromatic lens, which is used for adjusting the different working distances of the microscope. The transmission characteristics of the filter were chosen to block as less as only the respective wavelength of the laser to achieve the best possible image representation during the procedure (Fig. 1). Before the procedure, a white balancing procedure is performed to minimize shift of the colors represented in the digital binocular due to the installed filter and to adjust the surrounding illumination on the imaging chain of the microscope. In addition, adjustment of the surgeon’s vision on the OLED displays in the digital binocular was done with a microscope integrated test image and the diopter adjustment ring. The microscope provided exactly the same image with the same characteristics for the surgeon and the co-observers.

**Fig. 2:**
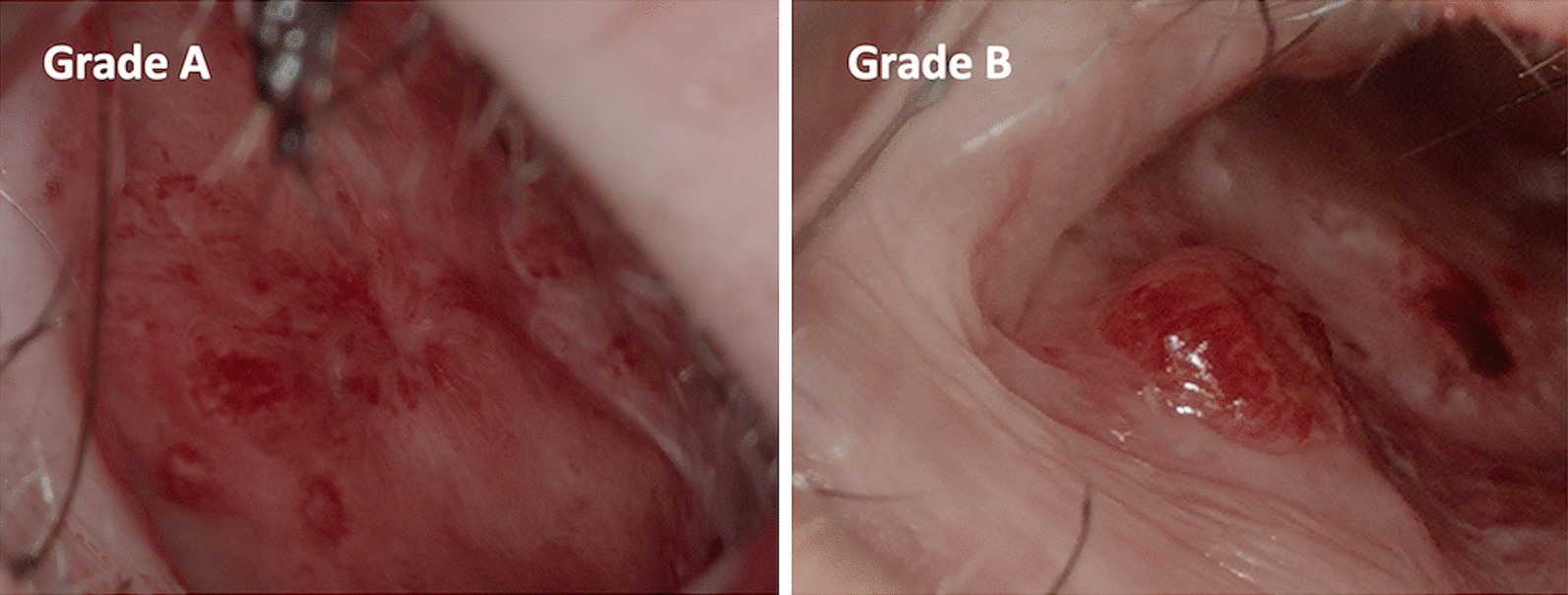
Classification of endonasal telangiectasias in HHT; **a** Grade A: presence of only flat telangiectasias in the mucosa level, **b** Grade B: (additional) presence of raised berry or wart-like telangiectasia spots

The recorded photos and videos during the treatment were used to classify the endonasal manifestations according to their 3D-morphology. Analogue to the Paquet classification of esophageal varices [18], endonasal manifestations were classified as follows (Fig. 2):

- Grade A: presence of only flat telangiectasias in the mucosa level.
- Grade B: (additional) presence of raised berry or wart-like telangiectasia spots.

The classification was performed independently by three persons of the author group. The average of this estimation was used for further analysis.

### Severity of epistaxis, quality of life

Severity of epistaxis was evaluated by epistaxis severity score (ESS) [19]. This score ranges from 0 to 10, while lower scores correlate with mild epistaxis and higher scores correlate with severe epistaxis. To quantify the quality of life, patients were asked to mark a linear visual analogue scale (VAS), indicating the current quality of life in respect to HHT [20]. The VAS was ranged from 0 (the worst conceivable quality of life) to 10 (the best conceivable quality of life) [20]. VAS and ESS were evaluated prior to the treatment.

### Determination of plasma VEGF and Haemoglobin

Peripheral venous blood samples were taken from patients prior to the procedure within regular monitoring of the blood count. Haemoglobin levels were measured by routine laboratory procedures. VEGF-plasma levels were determined by quantitative sandwich enzyme immunoassay technique (ELISA).

### Statistical analysis

Data analysis was performed using R (R Core Team, R: A Language and Environment for Statistical Computing, R Foundation for Statistical Computing, 2017; R version 3.6.1 (2019-07-05)). Unless otherwise stated, further analysis was performed with built-in packages and functions from the CRAN package tidyverse. The correlations were investigated using Spearman’s rank correlation coefficients (rs) and Wilcoxon signed-rank test. P values < 0.05 were considered to be statistically significant. Results are expressed as mean ± SEM (standard error of the mean).

## Results

### Descriptive data of the patients

28 patients, including 13 males and 15 females, were enrolled in this study. Patient’s age ranged from 26 to 82 years, with an average age of 56.6 ± 16.1 years. According to the microscopic image of endonasal telangiectasias, 11 patients (39.3 %) were classified as grade A and 17 patients (60.7 %) were classified as grade B. The average age of the grade A patients was 56.1 ± 16.9. The average age of the grade B patients was 56.9 ± 13.8. The screening tests for extranasal manifestations including contrast enhanced MRI of brain, gastroscopy, colonoscopy, contrast enhanced sonography of liver and echocardiography were recommended to all patients. 23 Patients had been examined for extranasal manifestations of HHT, including cerebral, gastrointestinal, hepatic and pulmonary arteriovenous malformations. The occurrence of extranasal manifestations was confirmed in 16 (69.5 %)of these patients.

### Classification of endonasal manifestation of HHT and clinical parameter

**Fig. 3:**
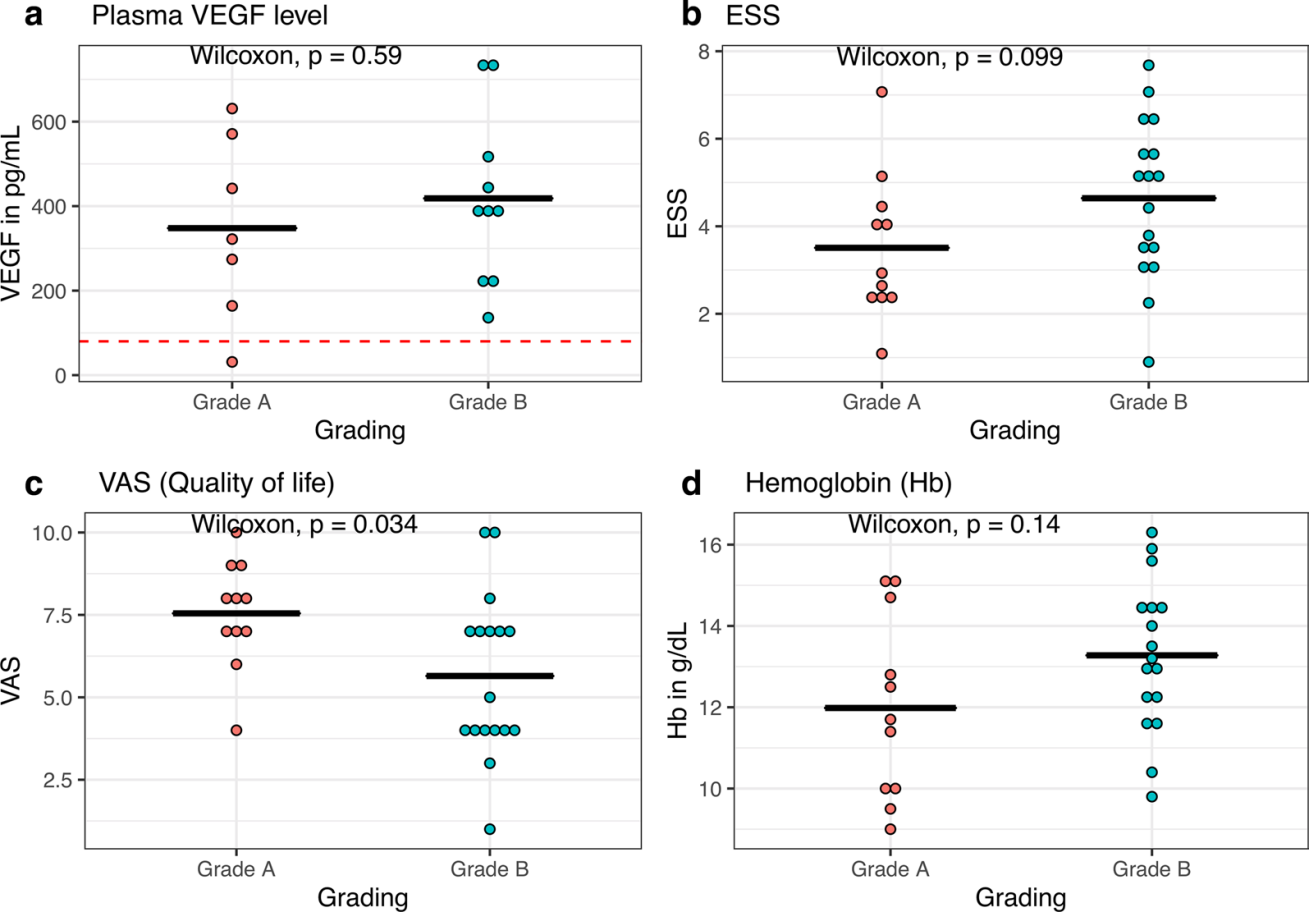
Classification of endonasal manifestation of HHT and clinical parameter: Grade A and B vs. **a** Plasma VEGF level, **b** epistaxis severity score, **c** Quality of life by VAS and **d** Haemoglobin. Data are presented as mean ± SEM. Red line represents normal value of VEGF

HHT patients in this study presented a mean value of plasma VEGF of 389.37 ± 204.23 pg/ml. There was no difference between the plasma VEGF level in group A and B (Fig. 3a), while the plasma VEGF level in 27 patients was significantly higher than plasma VEGF in healthy individuals with a normal value < 80 pg/ml [15, 25]. Plasma VEGF level in just one patient was with 31.3 pg/mL under the normal value, who was pregnant at the time of examination. There was no statistically significant difference with respect to the Epistaxis Severity Score (Fig. 3b), but there was a trend toward higher ESS values in group B. Quality of life in patients with grade B of endonasal manifestations was significantly lower compared to that in patients with grade A manifestations (Fig. 3c). Haemoglobin levels were not different in both groups (Fig. 3d). Extranasal manifestations of HHT were diagnosed in 6 out of 10 patients with grade A of endonasal telangiectasias and in 11 out of 13 patients with grade B lesions.

### Correlation of plasma VEGF level and clinical parameter

No correlation was found between plasma VEGF level and severity of epistaxis (ESS-Score) as well as quality of life (VAS). Haemoglobin levels showed also no correlations with plasma VEGF level (Table 1).

**Table 1:** Correlation of plasma VEGF level to severity of epistaxis (ESS), quality of life (VAS) and haemoglobin

Correlation of plasma VEGF level to:	
ESS	r_s_ = 0.19 p = 0.47
VAS	r_s_ = − 0.29 p = 0.25
Haemoglobin	r_s_ = 0.022 p = 0.93

r_s_: Spearman’s rank correlation coefficients, p: p value

**Fig. 4:**
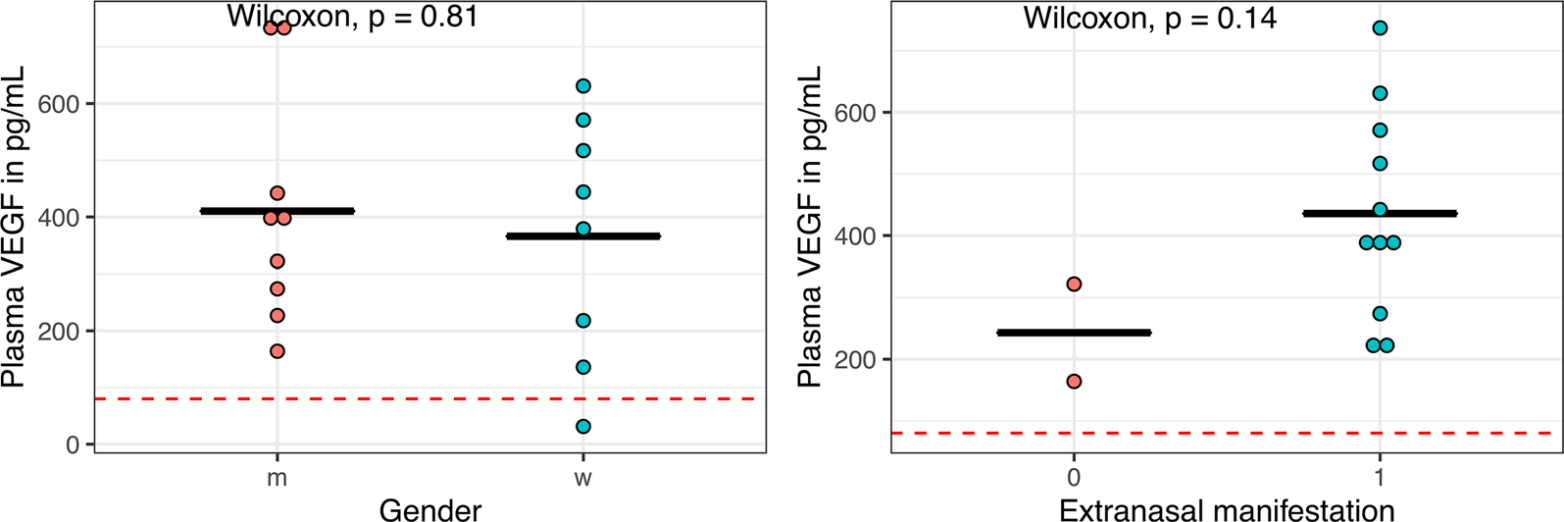
**a** Plasma VEGF level in male and females, **b** Plasma VEGF level and extranasal manifestation of HHT. Red line represents normal value of VEGF

A significant difference between plasma VEGF level in males and females could not be detected (Fig. 4a). 13 patients were assessed for both plasma VEGF levels and extranasal manifestations of HHT. Only 2 of these patients did not show any extranasal involvement; the plasma VEGF level in these 2 patients was lower compared to the other 11 patients (Fig. 4b).

## Discussion

### Classification of endonasal manifestation of HHT and clinical parameters

Despite various available conservative and procedural methods, treatment of recurrent epistaxis is still a challenging aspect of HHT. Most available treatments stop or reduce the nasal symptoms only temporarily and recurrence of epistaxis remains anticipated. The development of endonasal angiodysplastic lesions in HHT patients is a dynamic process influenced by many factors. Untreated telangiectasias increase in size and show an increased tendency to merge [12]. New telangiectatic lesions develop in previously untreated areas of the mucosa. Physical properties of respiratory airflow may induce angiogenesis by causing subacute inflammation due to nasal dryness [12]. The density of nasal telangiectasias increases with advancing age [12, 21]. Development of endonasal telangiectasias is also affected by hormonal changes [22]. Understanding morphology and dynamic of endonasal telangiectasias and its correlation to clinical symptoms is imperative for optimal management of the epistaxis in HHT.

Up to now, nasal distribution and morphology of telangiectasias has been addressed by relatively few studies. One of the most probable reasons could be due to objective difficulties in precise and complete inspection and recording of endonasal manifestations. In the present study, we used a digital 3D microscope with a considerably better depth impression compared to conventional analogue microscopes. This allows a more precise assessment of distribution and 3D morphology of the endonasal lesions during the procedure. Moreover, the microscope records the respective images for the left and the right eye separately. These recorded digital images can be visualized offline in three dimensions for postoperative assessments. For example, the images could be presented to an independent Panel for a more objective evaluation. Moreover, the photos and videos can also be analysed over the course of time, allowing the study of the 3D dynamics of endonasal HHT manifestations.

The microscope can enhance structures by image enhancement. Therewith it is possible, to highlight the vessel system. This technology could be also used in the future to match images by computer vision during a time series and to evaluate the same positions during therapy. This would allow an even more detailed insight into the changes in vascularization in patients with telangiectasia.

Mahoney et al. [13] classified 2006 the intranasal vasculature patterns in three types: (I) isolated punctate telangiectasias or individual small AVM; (II) diffuse interconnecting vasculature with “feeder” vessels; and (III) large solitary AVM, which may be associated with scattered telangiectasia. They observed a higher epistaxis severity (evaluated using Rebeiz classification[23]) in type II patients, associated with an increased need for transfusions and a poorer response to the Nd:YAG laser treatment. Pagella et al. [24] studied 2016 the correlation of severity of epistaxis with nasal telangiectasias in HHT. They observed no significant correlation between the presence of confluencing lesions and the severity of epistaxis nor the need for increased blood transfusions, unlike Mahoney. They proposed a new classification and defined three types of patients: “punctate pattern,” when all subsites involved present only flat punctate telangiectasias; “large pattern,” when all subsites involved present only prominent large lesions; and the “mixed pattern,” when both flat punctate and prominent large telangiectasias are present in the nasal cavity. Pagella et al. observed a significantly higher frequency of epistaxis in patients presenting the “large pattern”. They also reported an increased intensity of epistaxis with increases in number of nasal subsites involved. In the present study, we classified endonasal manifestation of HHT patients into two grades, reflecting the 3D morphology: grade A, with presence of only flat telangiectasias restricted to the mucosal level, and grade B, with presence of exophytic, berry or wart like lesions. These grades can be considered to be similar, respectively, to “punctate pattern” and “large and mixed pattern” following the classification proposed by Pagella and co-workers. We observed that patients with grade B nasal manifestations tended to suffer from more severe epistaxis. The data of the present study show a significantly better quality of life by VAS in grade A patients. This is in line with the results of the cited studies. We observed no differences in the Haemoglobin levels. Haemoglobin values can be affected by numerous factors such as gender, dehydration and comorbidities, which could easily influence the statistical results in a relatively small sample, as in this study. Therefore, the reproducibility of the current results needs to be further investigated. Considering the experiences from these studies, defining a classification for nasal manifestation of the HHT may require analysis of both morphology and distribution of telangiectasias. For this purpose, the next step could be mapping of nasal mucosa using digital microscopy with the ability of depth measurement. This could involve analysis of various factors, such as 3D morphology of single lesions, confluence between single lesions, number of nasal subsites involved and density of lesion in these sites. That is why we suggest a new classification according to localization and morphology of HHT lesions. The appropriate addressing of these aspects needs more advanced microscopic tools. Future improvements in imaging technology (image enhancement, narrow band imaging, etc.) could improve the sensitivity of the classification.

### Correlation of plasma VEGF level and clinical parameter

HHT is caused by mutations in several genes of the transforming growth factor (TGF)-β superfamily pathway, such as vascular endothelial growth factor (VEGF) [8, 9]. Recent progress in the therapy of HHT has been made using drugs that target different points of these signalling pathways. One of the most effective treatment modalities in HHT is systematic or local therapy with bevacizumab (anti-VEGF antibody) [8]. According to the previous studies the reference range of plasma VEGF in healthy individuals is < 80 pg/ml [15, 25]. Plasma VEGF levels have been found to be elevated significantly in HHT patients and are correlated directly with higher local expression of VEGF in nasal mucosa [9, 15]. It has also been shown that plasma VEGF levels are positively correlated with the degree of bleeding reductions after local submucosal bevacizumab treatment [15, 16, 26]. In the present study, 17 of 18 patients presented elevated plasma VEGF levels compared to the reference values for healthy subjects. The mean plasma VEGF level was also considerably higher than 80 pg/dl. We observed no significant gender specific difference for plasma VEGF level. Liu et al. [16] showed in 2020 moderate correlations between plasma VEGF and both VAS bleeding scores and the duration of moderate bleedings. Cirulli et al. [9] reported 2003 a positive correlation between plasma VEGF and frequency of epistaxis. We could not determine any significant correlation between plasma VEGF level and haemoglobin. However, plasma VEGF level tends to be noticeably higher in the majority of patients with extranasal manifestation of HHT than in patients without extranasal manifestation. Additionally, plasma VEGF levels in patients with grade B of endonasal telangiectasias tended to be higher than in grade A patients. This is in compliance with the results of epistaxis severity and quality of live assessment; a higher plasma VEGF and grade B telangiectasias, both seemed to be associated with more severe nasal bleeding symptoms (Additional file 1).

### Limitations

A major limitation in the present study is the small sample size compromising statistical power.

One more limiting issue, that should be investigated in the future studies, is the reliability of the categorisation of HHT lesions. Although the digital 3D microscopy allows an objective or semi-objective classification of telangiectasias using offline imaging, the reliability of categorisation should be supported by evidence of high inter-rater agreement and test-retest reliability.

A further relevant confounding factor could be the possible intra-individual variability of plasma VEGF level. Therefore, the results of study do not allow a definitive interpretation and should warrant further investigation in larger prospective studies in HHT patients.

## Conclusions

Digital microscopy is helpful to classify and document intranasal HHT lesions. A standard classification for endonasal manifestation of HHT can give an indication for severity of clinical symptoms and assists in the evaluation of the treatment efficacy. It requires comprehensive analysis of morphology and distribution of telangiectasias in larger prospective studies.

## Supplementary Information

**Additional file 1**. Raw data and patients characteristics are available by the authors.

## Data Availability

Please contact author for data requests.
